# Test–Retest Reliability and Concurrent Validity of Photoplethysmography Finger Sensor to Collect Measures of Heart Rate Variability

**DOI:** 10.3390/sports13020029

**Published:** 2025-01-22

**Authors:** Donald W. Rogers, Andreas T. Himariotis, Thomas J. Sherriff, Quentin J. Proulx, Megan T. Duong, Sabrina E. Noel, David J. Cornell

**Affiliations:** 1 Health Assessment Laboratory, University of Massachusetts Lowell, Lowell, MA 01854, USA; donald_rogers@student.uml.edu (D.W.R.); andreas_himariotis@student.uml.edu (A.T.H.); t.sherriff7997@gmail.com (T.J.S.); quentin_proulx@student.uml.edu (Q.J.P.); megan_duong@student.uml.edu (M.T.D.); sabrina_noel@uml.edu (S.E.N.); 2 Department of Physical Therapy and Kinesiology, University of Massachusetts Lowell, Lowell, MA 01854, USA; 3 Department of Public Health, University of Massachusetts Lowell, Lowell, MA 01854, USA

**Keywords:** autonomic nervous system, electrocardiography, heart rate monitor, field-based monitoring, sport science

## Abstract

The purpose of the current study was to determine the test–retest reliability and concurrent validity of a photoplethysmography (PPG) finger sensor when collecting heart rate variability (HRV) metrics in reference to electrocardiography (ECG) and heart rate monitor (HRM) devices. Five minutes of R-R interval data were collected from 45 participants (23 females; age: 23.13 ± 4.45 yrs; body mass index: 25.39 ± 4.13 kg/m^2^) in the supine and seated positions in testing sessions 48 h apart. *Moderate-to-excellent* test–retest reliability of the HRV data collected from the PPG sensor was identified (ICC_2,1_ = 0.60–0.93). Additionally, similar standard errors of the mean, coefficient of variation, and minimal detectable change metrics were observed across all devices. Statistically significant (*p* < 0.05) differences were identified in the HRV data between the PPG sensor and ECG and HRM devices; however, these differences were interpreted as *trivial-to-small* (*g* = 0.00–0.59). Further, the PPG sensor tended to only overestimate HRV metrics by <0.5 ms and *near perfect* relationships (*r* = 0.91–1.00) and *very large-to-near perfect* agreement (CCC = 0.81–1.00) were identified between collection methods. The PPG sensor demonstrated adequate test–retest reliability and concurrent validity in both the supine and seated resting positions.

## 1. Introduction

Heart rate variability (HRV) is the beat-to-beat variability in R-R interval length observed on an electrocardiogram [1]. HRV is used as a non-invasive measure of autonomic nervous system (ANS) function and balance [2]. From a simplistic perspective, greater HRV was historically associated with greater vagus nerve activity, while lower HRV was generally associated with less vagus nerve activity [3]. However, it is now understood that HRV reflects a confluence of neurocardiac function, including blood pressure, gas exchange, vascular tone, and gastrointestinal function [4]. For this reason, HRV has become a useful metric across multiple human health and performance disciplines over the decades [2]. Clinically, reduced resting HRV has been associated with cardiovascular mortality [5,6,7] and incident heart failure [8,9]. Sport scientists specifically use HRV metrics in a number of capacities [10], including to guide exercise prescription and progression, examine physiological adaptions, monitor training workload and post-exercise recovery, track overreaching and prevent overtraining, and predict and enhance sports performance [11].

The historical standard of short-term HRV data collection is at least 5 min of stationary supine recording using an electrocardiography (ECG) device [12]. However, other positions of data collection, such as seated [13,14], have been used in both the scientific literature and among practitioners. Ultra short-term recordings (e.g., <5 min) have also been proposed by researchers [15,16,17] and utilized by practitioners [18,19], but questions regarding the physiological validity of such HRV recordings still exist [20], resulting in 5 min recordings still being considered the criterion of choice [21].

The use of ECG technology and the subsequent need of third-party software packages to process R-R interval data has previously limited the widespread application of HRV metrics in many applied or field-based settings [22]. However, photoplethysmography (PPG) technology is now being used in new ways to potentially provide a more feasible method of HRV data collection [2]. Specifically, PPG is an optical technique that can detect the changes in blood volume in the vasculature at the recording site [23]. PPG sensors consist of a light emitting diode and a light detecting diode on opposite sides of the tissue bed [24]. As the amount of light detected by the sensor changes, due to changes in blood volume, a pulsatile waveform is produced. Each peak in the waveform indicates a heartbeat, and due to the sensitivity of the PPG sensor, it can theoretically be used to measure HRV by characterizing the variability in the peaks of the pulse waveform, known as pulse rate variability [24]. PPG technology is much easier and more convenient to use for daily collection of HRV metrics since all that is required is a sensor placed over the peripheral pulse location to measure HRV [22]. Such locations include the earlobe, wrist, and finger, including via the use of a smartphone camera [22]. Comparatively, ECG devices require the placement of ECG electrodes directly onto skin for the collection of R-R interval data used to derive HRV measures, which are typically not available to the general population and practitioners. Similarly, chest heart rate monitors (HRM) require the direct contact of the monitor onto the participant’s chest for the collection of R-R interval data, which may not be feasible for all individuals in all settings (e.g., clinics, upon wakening, athletic teams, etc.).

New PPG sensor technology and smartphone applications have now allowed for the processing of R-R interval data and deriving of HRV measures in real time [25,26]. Thus, these new technologies that allow instantaneous calculation of HRV metrics has increased the feasibility of HRV data collection for daily monitoring of HRV metrics by the individual in different collection settings [22,27]. However, given the differences in sampling frequencies, light diodes, collection locations, and software associated with these technologies [27], the validity of each new device must be established before it can be confidently utilized to assess HRV. Furthermore, although HRV itself presents with day-to-day fluctuations, it is still important to determine device test–retest reliability relative to other criterion methods when establishing the concurrent validity of the device [28]. Specifically, it is important to determine if these new technologies demonstrate similar stability in order to be confident they can be used for longitudinal HRV collection scenarios [29].

Few studies have concomitantly evaluated both the test–retest reliability and validity of a PPG sensor to collect HRV [30,31,32,33], and none have simultaneously compared both the reliability and validity in reference to two different criterion devices in two different positions. While ECG is the gold-standard collection method, HRMs have been the long-standing technology for HRV collection in field-based settings, and thus, are still useful industry-standard comparison criterion to validate use of PPG sensors in field-based situations [34]. A new fingertip PPG device, known as the CorSense (Elite HRV, Asheville, NC, USA), has been introduced to the consumer market, but the test–retest reliability and validity of this specific device has not been established. Therefore, the purpose of the current study was to determine both the test–retest reliability and concurrent validity of a new PPG finger sensor paired with a smartphone application when collecting HRV data in reference to gold-standard ECG and previously validated industry-standard HRM devices in both the supine and seated position. It was hypothesized that adequate test–retest reliability and concurrent validity would be observed for HRV metrics collected via the PPG finger sensor in the supine and seated positions. Preliminary results from this study have been published in abstract format only [35,36].

## 2. Materials and Methods

### 2.1. Participants

Participants were recruited to participate in this study via flyers, emails, and word-of-mouth throughout the university campus. Eligibility criteria included adults at least 18 years old and willing to provide informed consent. Anyone who had been previously diagnosed with known cardiovascular disease, cardiac arrythmia, or currently taking any medications that were prescribed to control heart rate or heart function were excluded from the study. All study protocols were approved by the Institutional Review Board (IRB) at the University of Massachusetts Lowell (IRB Protocol #: 19-163-COR-XPD). In addition, all procedures were in accordance with the principles of the World Medical Association’s Helsinki Declaration. Participants were given the opportunity to ask any questions before providing informed consent. This study adhered to STROBE guidelines and verified utilizing a checklist available in the Supplementary Material (Supplementary Table).

### 2.2. Protocol

After obtaining informed consent, anthropometric data were collected according to guidelines from the American College of Sports Medicine [37]. Body mass (kg) and height (cm) data were collected using a Detecto 2391 physician mechanical beam scale and stadiometer (Detecto, Inc., Webb City, MO, USA) to the nearest 0.1 kg and 0.5 cm, respectively.

All data were collected across two testing sessions conducted 48 h apart at the same time of day. Participants refrained from smoking tobacco (or any other tobacco product) and alcohol and caffeine intake for the four hours preceding each testing session, as well as refraining from vigorous physical activity for 48 h prior to each testing session [38]. Participants were also asked to use the bathroom before data collection [39].

**Figure 1:**
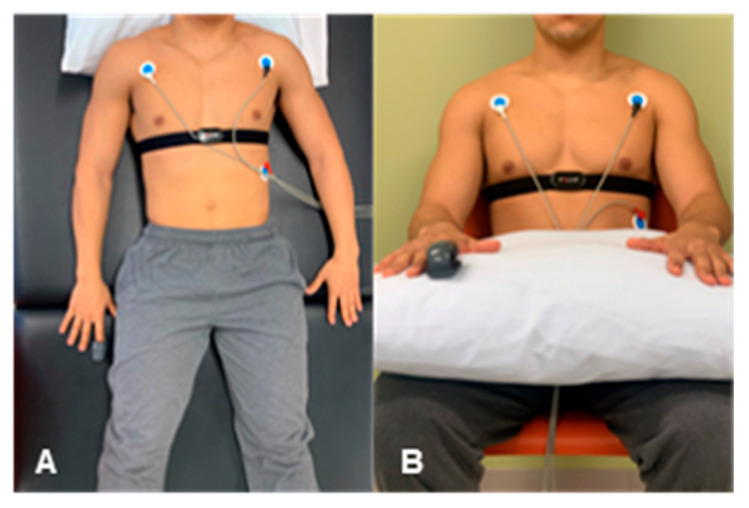
Testing protocol in both the supine (**A**) and seated (**B**) positions.

The testing protocol did not differ across testing sessions. Prior to any data collection in either position, 5 min of stabilization in both supine and sitting occurred to minimize the influence of body position change, which is consistent with resting HRV data collection procedures [40,41]. After each stabilization, 5 min of R-R interval data were then collected using a spontaneous breathing pattern, first in supine (Figure 1A), and secondly in the seated (Figure 1B) position. Participants were given no breathing instructions to be consistent with other field-based HRV data collection methods [42].

R-R interval data were collected utilizing a 3-lead electrocardiogram (ECG) [12] and Cosmed Heart Rate Variability software Version 5.515962 (Cosmed Inc., Rome, Italy; sampling rate: 1000 Hz) with electrodes placed under the clavicles of their right and left shoulders and left abdomen [43]. To reduce impedance, the skin of each participant was cleaned and abraded using alcohol prep pads. In addition, a Polar H10 HRM (Polar Electro, Kempele, Finland; sampling rate: 1000 Hz) was placed at the level of the xiphoid process and paired to a Polar V800 watch (Polar Electro, Kempele, Finland). This monitor currently serves as an industry standard for HRV data collection as it has been previously validated in reference to ECG HRV data collection methods [44]. Lastly, a CorSense PPG finger sensor (Elite HRV, Asheville, NC, USA; sampling rate: 500 Hz) was placed on the right index finger (Figure 1) and paired with the Elite HRV smartphone application (Elite HRV, Asheville, NC, USA) [45].

Heart rate (HR) and HRV metrics derived from the R-R interval data collected by the PPG finger sensor were processed entirely within the Elite HRV smartphone application [45]. However, both HR and HRV metrics derived from R-R interval data collected by the ECG and Polar H10 HRM were downloaded to an external laptop. These data were then processed via Kubios HRV 3.5.0 software (Kubios, Ltd., Kuopio, Finland) using an automatic artifact correction threshold [46], which has been previously demonstrated to derive R-R interval data in an equivalent manner to the Elite HRV smartphone application [45]. The root mean square of successive heartbeat differences (RMSSD) and the standard deviation of normal R-R intervals (SDNN) were the time-domain parameters utilized as both parameters are commonly utilized during short-term recordings in field-based HRV data collection [34,47].

### 2.3. Statistical Analysis

Examination of the histograms and Q-Q plots indicated that the HRV metrics of RMSSD and SDNN violated the assumption of normality. As a result, the natural log of RMSSD (lnRMSSD) and the natural log of SDNN (lnSDNN) were calculated and utilized in all statistical analyses, which is consistent with the scientific literature [48]. Neither R-R interval data nor HR data violated the assumption of normality.

To determine the test–retest reliability of the HRV metrics (R-R intervals, HR, lnRMSSD and lnSDNN) collected by the ECG, Polar H10 HRM, and PPG finger sensor devices, a variety of statistical approaches were utilized [49]. Specifically, two-way random intraclass correlation coefficients (ICC_2,1_) and the standard error of the measure (SEM) were utilized to measure the relative and absolute reliability of the HRV metrics across both testing sessions, respectively [50]. Using the SEM, the minimal detectable change (MDC) was calculated at a 95% confidence level [51].

$$\mathrm{MDC}=\mathrm{SEM}\times 1.96\times \sqrt{2}$$

The coefficient of variation (CV) was calculated by dividing the average standard deviation (SD) by the average mean across both testing sessions and expressed as a percentage [49].

$$CV=\left(\frac{\left[\frac{\left(\mathrm{SD}\;\mathrm{testing}\;\mathrm{session}\;1\;+\;\mathrm{SD}\;\mathrm{testing}\;\mathrm{session}\;2\right)}{2}\right]}{\left[\frac{\left(\mathrm{Mean}\;\mathrm{testing}\;\mathrm{session}\;1\;+\;\mathrm{Mean}\;\mathrm{testing}\;\mathrm{session}\;2\right)}{2}\right]}\right)\times 100$$

Several statistical approaches were also utilized to determine the concurrent validity of the HRV metrics collected by the PPG sensor in reference to validated collection methods (i.e., ECG and Polar H10 HRM) [52].

Specifically, paired *t*-tests and Hedges’ *g* effect sizes were utilized to determine the absolute agreement [52] and magnitude of the differences [53] between the PPG finger sensor and the ECG and Polar HRM. Relative agreement was assessed utilizing Lin’s concordance correlation coefficients (CCC) [54] and bivariate Pearson correlations (*r*), and coefficients of determination (*R*^2^) identified associations and level of variance explained by the PPG finger sensor compared to both criterion devices [55].

Bland–Altman plots were also constructed to examine agreement between the PPG finger sensor and the ECG and Polar HRM by plotting the differences between devices (y-axis) vs. the means between devices (x-axis) [56]. Specifically, the mean bias and 95% confidence intervals (95%CI) limits of agreement (LoA) around the mean bias were calculated based on the standard error of the mean differences $\left(\sqrt{{SD}^{2}/n}\right)$, which represented accuracy of the PPG finger sensor relative to the criterion [57]. Ninety-five percent CIs were also calculated around each upper and lower band of the LoA based on the standard error of the LoAs $\left(\sqrt{\left(3\times {SD}^{2}\right)/n}\right)$, which represented precision of the PPG finger sensor relative to the criterion. A regression line was then subsequently plotted against the means between devices and the differences between devices, which represented proportional error in the agreement between devices. Finally, percentage errors (PEs) were calculated to express the mean bias as a percentage by dividing the mean bias by the mean measure of the PPG finger sensor and either criterion [58].

$$\mathrm{PE}=\left(\;\frac{100\times [1.96\times \mathrm{SD}\;\mathrm{of}\;\mathrm{Bias}]}{\left[\frac{\mathrm{Mean}\;\mathrm{Criterion}\;+\;\mathrm{Mean}\;\mathrm{PPG}}{2}\right]}\right)$$

ICCs were interpreted as: *excellent*: ICC ≥ 0.90; *good*: 0.90 > ICC ≥ 0.75; *moderate*: 0.75 > ICC ≥ 0.5; *poor*: ICC < 0.5 [59]. Hedges’ g effect sizes were interpreted qualitatively as: *very large*: g ≥ 2.0; *large*: 2.0 > g ≥ 1.2; *moderate*: 1.2 > g ≥ 0.6; *small*: 0.6 > g ≥ 0.2; or *trivial*: g < 0.2 [60]. Pearson’s correlation coefficients (r) were interpreted as: *near perfect*: r ≥ 0.9; *very strong*: 0.9 > r ≥ 0.70; *strong*: 0.70 > r ≥ 0.50; *moderate*: 0.50 > r ≥ 0.30; *small*: 0.30 > r ≥ 0.10; or *trivial*: r < 0.10 [61]. Lin’s CCC were interpreted as: *near perfect*: CCC ≥ 0.90; *very large*: 0.90 > CCC ≥ 0.70; *large*: 0.70 > CCC ≥ 0.5; *moderate*: 0.5 > CCC ≥ 0.3; *small*: 0.3 > CCC ≥ 0.10; *trivial*: CCC < 0.10 [60]. Lastly, PEs were interpreted using a clinically accepted threshold of < 30% [58]. An alpha level of 0.05 was used to determine statistically significant differences in HRV metrics collected by the finger sensor and either the ECG or HRM. All statistical analyses were conducted using Microsoft Excel (Microsoft Cor., Redmond, WA, USA) and SPSS v28 statistical software (IBM Corp., Armonk, NY, USA).

Based on recommendations from the scientific literature, a near perfect correlation coefficient (*r* > 0.90) should be observed to determine reliability and validity [62]. Therefore, based on an expected effect size of ρ = 0.90, an a priori analysis was conducted using G*Power 3.9.1.7 software (Heinrich-Heine-Universitat Dusseldorf), which determined that a sample size of 8 participants was required to achieve 95% power (1 − β) when identifying statistically significant associations at the 95% confidence level (α = 0.05) between devices [63].

## 3. Results

A total of 47 participants (25 females, 22 males) were initially recruited through the university campus to participate in this study. However, two participants decided to withdraw from the study before any data was collected, leaving a sample size of 45 participants (80% White or Caucasian, 11% Asian, 4% Hispanic, 2% Black or African American, and 2% multiracial), which is consistent with other HRV validity studies in the scientific literature [25,30,31,32,33,34,64,65,66,67].

**Figure 2:**
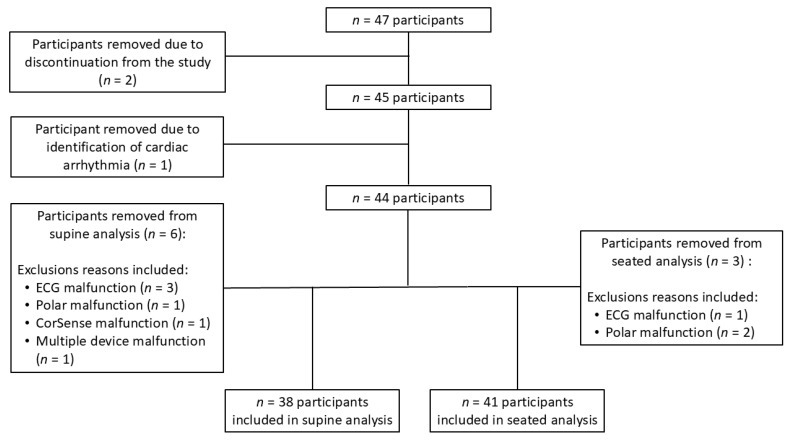
Flow chart of initial recruitment, participants discontinued from study, and participants excluded from final analyses.

A cardiac arrythmia was observed during data collection for one participant, and as a result, they were excluded from analyses, leaving 44 participants for initial analyses (age: 23.13 ± 4.45 yrs; height: 170.21 ± 9.08 cm; body mass: 74.14 ± 16.50 kg) (Figure 2). In addition, six people were removed from the supine analyses due to three ECG device malfunctions, one PPG device malfunction, one Polar HRM malfunction, and one multiple device malfunction (Figure 2) for a total of 38 participants (20 females, 18 males) included in the supine analyses (Table 1). Finally, three other participants were removed from the seated analysis due to one ECG device malfunction, and two Polar HRM device malfunctions (Figure 2) for a total of 41 participants (21 females, 20 males) included in the seated analyses (Table 1).

### 3.1. Supine

#### 3.1.1. Reliability

Good test–retest reliability of R-R interval data (ICC_2,1_ = 0.852–0.860), HR data (ICC_2,1_ = 0.847–0.855), and lnRMSSD data (ICC_2,1_ = 0.764–0.842) was identified among all devices between testing sessions. While good test–retest reliability of lnSDNN data was observed among the ECG and HRM devices (ECG: ICC_2,1_ = 0.815–0.820), only moderate reliability was observed among the PPG sensor data between both testing sessions (ICC_2,1_ = 0.604). However, all devices demonstrated similar SEM, MDC, and CV values for R-R interval, HR, and all HRV data (Table 2).

#### 3.1.2. Validity

**Figure 3:**
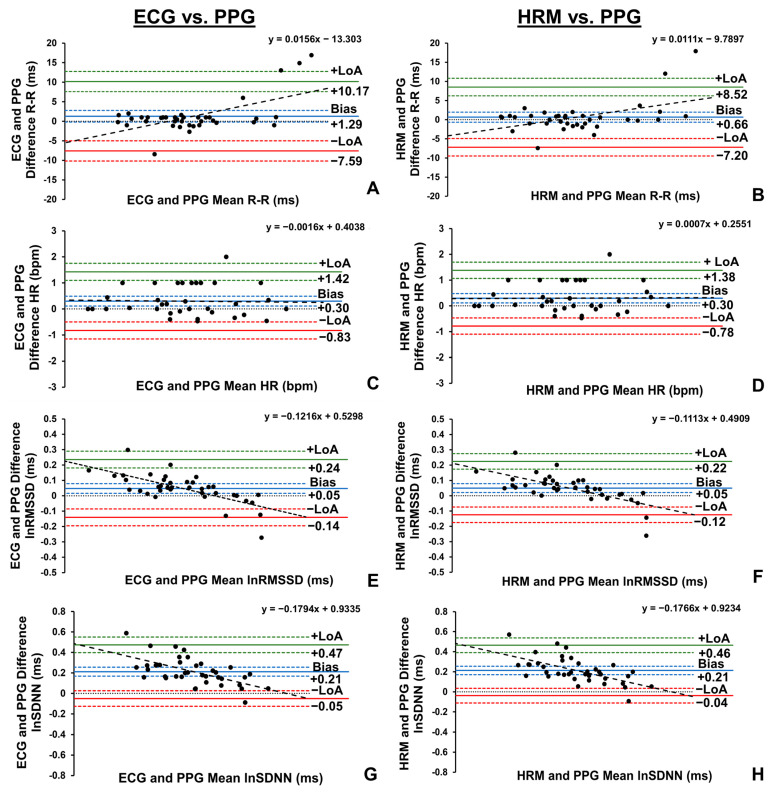
Bland–Altman plots demonstrating the mean bias, limits of agreement (LoA), respective 95% confidence intervals (CIs), and slope of linear regression line of R-R interval (**A**), heart rate (HR, **C**), natural log of root mean square of successive differences (lnRMSSD, **E**), and natural log of standard deviation of normal R-R intervals (lnSDNN, **G**) collected by photoplethysmography (PPG) sensor and electrocardiography (ECG) devices, and the mean bias, LoA, respective CIs, and slope of linear regression line of R-R interval (**B**), HR (**D**), lnRMSSD (**F**), and lnSDNN (**H**) collected by PPG sensor and chest heart rate monitor (HRM) devices in the supine position (*n* = 38).

*Trivial* and non-significant differences in the R-R interval data were observed between the PPG sensor and both the ECG (*p* = 0.087) and Polar HRM (*p* = 0.317) (Table 3), with a mean bias of 0.66 ms to 1.29 ms (Figure 3A,B). In addition, *near perfect* agreement and a *near perfect* relationship were identified between the PPG sensor and both the ECG (CCC = 1.000, 95%CI: 0.999, 1.000; *r* = 1.000, 95%CI: 1.000, 1.000, *R*^2^ = 1.000) and the Polar HRM (CCC = 1.000, 95%CI: 1.000, 1.000; *r* = 1.000, 95%CI: 1.000, 1.000, *R*^2^ = 1.000).

Statistically significant, but *trivial*, differences in the HR data were observed between the PPG sensor and both the ECG (*p* = 0.003) and Polar HRM (*p* = 0.002), with mean biases of 0.30 bpm (Figure 3C,D). Furthermore, *near perfect* agreement and *near perfect* relationships were observed between the PPG sensor and both the ECG (CCC = 0.999, 95%CI: 0.997, 0.999; *r* = 0.999, 95%CI: 0.998, 0.999, *R*^2^ = 0.998) and HRM (CCC = 0.999, 95%CI: 0.997, 0.999; *r* = 0.999, 95%CI: 0.998, 0.999, *R*^2^ = 0.998).

Although statistically significant differences in the lnRMSSD data were observed between the PPG sensor and both the ECG (*p* = 0.004) and the HRM (*p* = 0.001), these differences were interpreted as *trivial* with mean biases of 0.05 ms (Figure 3E,F). Furthermore, *near perfect* agreement and *near perfect* relationships were identified between the PPG sensor and the ECG (CCC = 0.981, 95%CI: 0.969, 0.999; *r* = 0.992, 95%CI: 0.985, 0.996, *R*^2^ = 0.984) and the HRM (CCC = 0.983, 95%CI: 0.971, 0.990; *r* = 0.993, 95%CI: 0.986, 0.996, *R*^2^ = 0.986).

Similarly, statistically significant differences in the lnSDNN data were observed between the PPG sensor and both the ECG (*p* < 0.001) and the HRM (*p* < 0.001), but these differences were considered *small* with mean biases of 0.21 ms (Figure 3G,H). Additionally, *very large* agreement and *near perfect* relationships were observed between the PPG sensor and the ECG (CCC = 0.861, 95%CI: 0.783, 0.913; *r* = 0.971, 95%CI: 0.945, 0.985, *R*^2^ = 0.943) and the HRM (CCC = 0.863, 95%CI: 0.786, 0.913; *r* = 0.974, 95%CI: 0.951, 0.987, *R*^2^ = 0.949).

### 3.2. Seated

#### 3.2.1. Reliability

Good test–retest reliability of R-R interval data (ICC_2,1_ = 0.862–0.875) and HR data (ICC_2,1_ = 0.877–0.885) were observed among all devices between testing sessions. Excellent test–retest reliability of lnRMSSD data (ICC_2,1_ = 0.926–0.929) and lnSDNN data (ICC_2,1_ = 0.907–0.909) were observed among the ECG and HRM devices between testing sessions, and good reliability of lnRMSSD data (ICC_2,1_ = 0.889) and lnSDNN data (ICC_2,1_ = 0.848) was observed from the PPG sensor. In addition, all devices demonstrated similar SEM, MDC, and CV values for all HRV metrics (Table 4).

#### 3.2.2. Validity

**Figure 4:**
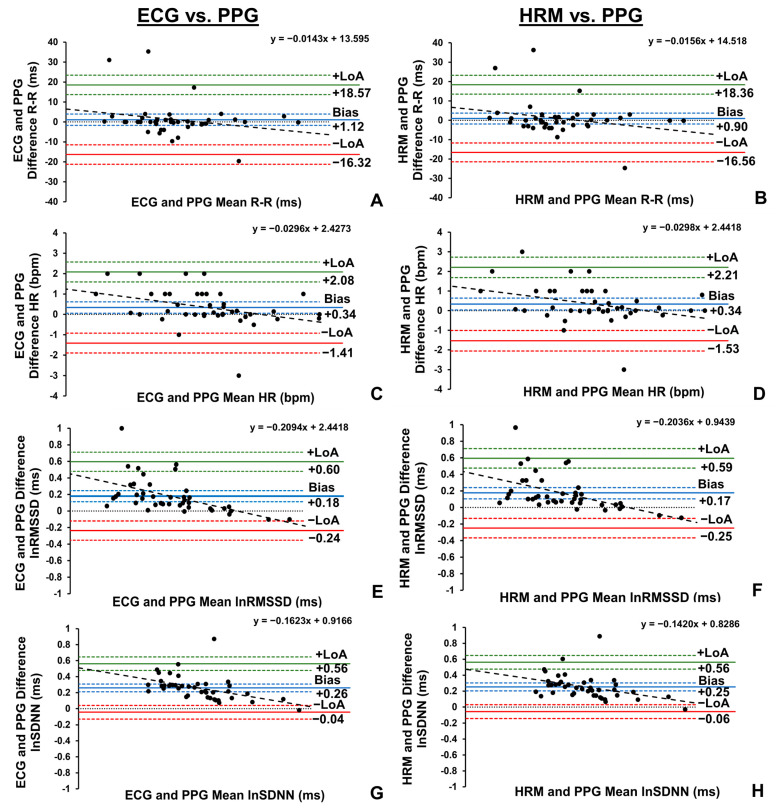
Bland–Altman plots demonstrating the mean bias, limits of agreement (LoA), respective 95% confidence intervals (CIs), and slope of linear regression line of R-R interval (**A**), heart rate (HR, **C**), natural log of root mean square of successive differences (lnRMSSD, **E**), and natural log of standard deviation of normal R-R intervals (lnSDNN, **G**) collected by photoplethysmography (PPG) sensor and electrocardiography (ECG) devices, and the mean bias, LoA, respective CIs, and slope of linear regression line of R-R interval (**B**), HR (**D**), lnRMSSD (**F**), and lnSDNN (**H**) collected by PPG sensor and chest heart rate monitor (HRM) devices in the seated position (*n* = 41).

Trivial and non-significant differences were observed in the R-R interval data between the PPG finger sensor and both the ECG (*p* = 0.424) and the HRM (*p* = 0.554) (Table 5) with mean biases between 0.90 ms and 1.21 ms (Figure 4A,B). Near perfect agreement and near perfect relationships were identified between the finger sensor and both the ECG (CCC = 0.998, 95%CI: 0.997, 0.999; *r* = 0.998, 95%CI: 0.997, 0.999, *R*^2^ = 0.996) and the HRM (CCC = 0.998, 95%CI: 0.997, 0.999; *r* = 0.998, 95%CI: 0.997, 0.999, *R*^2^ = 0.996).

In parallel, trivial, and non-significant differences in the HR data were observed between the PPG finger sensor and the HRM (*p* = 0.285) with a mean bias of 0.34 bpm (Figure 4D). However, statistically significant, but still trivial, differences were identified in the HR data between the PPG finger sensor and the ECG (*p* = 0.02) with a mean bias of 0.34 bpm (Figure 4C). In addition, near perfect agreement and near perfect relationships were identified between the finger sensor and both the ECG (CCC = 0.996, 95%CI: 0.993, 0.998; *r* = 0.997, 95%CI: 0.995, 0.999, *R*^2^ = 0.994) and HRM (CCC = 0.996, 95%CI: 0.993, 0.998; *r* = 0.997, 95%CI: 0.994, 0.998, *R*^2^ = 0.994).

Statistically significant differences were identified in the lnRMSSD between the PPG finger sensor and both the ECG (*p* < 0.001) and the HRM (*p* < 0.001), but these differences were interpreted as small, with mean biases between 0.17 ms and 0.18 ms (Figure 4E,F). Furthermore, very large agreement and near perfect relationships were identified between finger sensor and ECG (CCC = 0.887, 95%CI: 0.801, 0.926; r = 0.944, 95%CI: 0.897, 0.970, *R^2^* = 0.891) and HRM (CCC = 0.878, 95%CI: 0.801, 0.927; r = 0.939, 95%CI: 0.890, 0.968, *R*^2^ = 0.881).

Similarly, small, and statistically significant differences were observed in the lnSDNN between the PPG finger sensor and both the ECG (*p* < 0.001) and the HRM (*p* < 0.001), with mean biases between 0.25 ms and 0.26 ms (Figure 4G,H). Additionally, very large agreement and near perfect relationships were identified between the finger sensor and ECG (CCC = 0.806, 95%CI: 0.707, 0.874; r = 0.953, 95%CI: 0.912, 0.975, *R*^2^ = 0.908) and HRM (CCC = 0.806, 95%CI: 0.703, 0.875; r = 0.945, 95%CI: 0.899, 0.971, *R*^2^ = 0.893).

## 4. Discussion

The purpose of this study was to determine both the test–retest reliability and concurrent validity of the CorSense PPG finger sensor paired with the Elite HRV smartphone application in reference to gold-standard ECG and industry standard chest HRM devices in both supine and seated positions.

Moderate-to-excellent (ICC_2,1_ = 0.60–0.93) test–retest reliability was observed between testing sessions by the PPG sensor among R-R interval, HR, lnRMSSD, and lnSDNN data in both positions. In addition, similar SEM, CV, and MDC metrics were observed across devices in all measures and positions indicating adequate test–retest reliability of the PPG sensor. Specifically, the response stability of the CorSense PPG finger sensor was comparable to the gold standard ECG and industry standard chest HRM, which implies practitioners can track day-to-day changes in HRV metrics in a similar manner. Kunkels et al. [31] observed similar test–retest reliability of R-R interval data collected by a different PPG finger sensor (Ithelete) in a similar age population. However, adequate test–retest reliability was only observed for lnRMSSD in the supine position (ICC = 0.98), with the authors noting insufficient test–retest reliability in the seated position (ICC = 0.63). Speer et al. [30] observed adequate test–retest reliability of an external PPG finger sensor for collection of R-R interval and HR data in children (3–5 years old), but reliability was poor (ICC = 0.32–0.41) for lnRMSSD HRV metrics. Only one previous study observed moderate-to-good test–retest reliability of an ear-based PPG sensor to collect RMSSD HRV data in the seated position [32]. Thus, this current study is the first to observe adequate test–retest reliability of an external (i.e., non-smartphone camera) finger-based PPG sensor to collect HRV metrics in both the supine and seated positions.

In terms of absolute validity in the current study, although statistically significant differences were identified in both lnRMSSD and lnSDNN metrics in the supine and seated positions in comparison to both criterion devices, these differences were considered to be trivial-to-small (*g* = 0.09–0.59) in nature. Additionally, any differences identified in the R-R interval data were not statistically significant and considered trivial (*g* = 0.00–0.03), and any statistically significant differences in HR data were all interpreted as trivial (*g* = 0.03). Therefore, due to the trivial-to-small differences identified and all the PEs below the clinically acceptable cutoff of 30% (0.84–11.00%) [58], acceptable absolute concurrent validity of the CorSense PPG finger sensor paired with the Elite HRV smartphone application was determined when compared to both the ECG and HRM criterion devices.

In general, the PPG sensor in the current study tended to overestimate the R-R interval, HR, and HRV data in both positions, which is similar to other findings in the scientific literature [27]. This overestimation was at most around one ms or one bpm, and thus, the likely explanation for any differences between the PPG sensor and the criterions being considered trivial-to-small. Speer et al. [30] identified similarly trivial differences in both R-R interval and HR data of a PPG finger sensor in reference to the Polar H10 chest HRM in children (3–5 years old) in the seated position; however, moderate differences were identified in both lnRMSSD and SDNN HRV metrics. This is noteworthy as the finger PPG sensor device the authors examined sampled data at the same frequency (500 Hz) as the PPG finger sensor in the current study. Coupled with the aforementioned test–retest reliability results, it is possible that the reliability and validity of a PPG finger sensor is weaker among children compared to young adults. However, this interpretation is limited, as Speer et al. [30] did not compare the PPG finger sensor to a gold standard ECG.

In regards to other finger PPG devices, Wong et al. [65] noted no statistically significant differences in comparison to ECG for R-R interval data and HR data, but statistically significant differences were noted in RMSSD data in the supine position. Esco et al. [66] similarly identified significant differences in lnRMSSD data regarding the seated position when using a finger PPG sensor device in comparison to an ECG device, but these difference were also considered trivial in magnitude.

When examining relative validity in the current study, very strong-to-near perfect agreement (CCC = 0.81–1.00) and near perfect relationships (*r* = 0.91–1.00) were identified in the R-R interval, HR, lnRMSSD, and lnSDNN data between the PPG sensor and both the ECG and HRM. Bland–Altman analysis revealed high levels of agreement between the PPG sensor and both criterion devices. As such, findings of the current study indicate adequate relative validity of the PPG sensor in both the supine and seated positions. Similarly, previous research has also identified high levels of agreement [31,64,65,66,67] and strong associations [64,65,66,67] between finger PPG-derived HRV and ECG-derived HRV data. However, most previous research did not include multiple device criterion (i.e., ECG and HRM) and/or multiple collection positions (i.e., supine and seated). Therefore, our study builds off the previous literature by determining the concurrent validity of a PPG sensor in a larger variety of collection contexts, which is important given the variety of HRV collection methods employed by practitioners.

Several studies have also identified good test–retest reliability [33] and adequate validity [25,33,34] of finger-based PPG using a smartphone camera. However, given the ever-changing smartphone camera technology and differences in cameras between smartphones, it is difficult to compare results of the current study to other devices. Further, researchers and practitioners should also be cautious about the smartphone camera method of collecting HRV data, as the reliability and validity could theoretically differ between smartphones. In contrast, the device used in the current study remains constant, and instead, pairs with different smartphones. This could theoretically allow for better translation of finger PPG sensor technology across different researchers and in an interdisciplinary fashion among practitioners. Notably, this PPG device may be useful for any practitioner interested in tracking longitudinal measures of HRV, such as sport scientists, strength and conditioning coaches, sport psychologists, and physical therapists, athletic trainers, and other rehabilitation staff. This is particularly relevant, as even though a chest strap HRM can be used in field-based setting, it is still cumbersome to use, making daily monitoring burdensome.

Finally, to minimize the influence of breathing, the RMSSD metric was chosen as one of the primary HRV measures, as this metric is known to be less influenced by respiratory influences [68]. However, it is known that respiration patterns can influence short-term resting SDNN collection [69]. This may explain the slight attenuation in reliability and validity of lnSDNN data compared to lnRMSSD data observed in the current study. This could be particularly important for practitioners collecting HRV in field-based settings where controlling the breathing patterns of individuals is difficult.

### Limitations

There were several limitations of the current study that should be noted. First, the sample population were young (23.13 ± 4.45 yrs) and healthy, which could limit the applicability of the findings to other populations. Some previous research has noted attenuation of PPG-derived HRV data specifically in healthy pediatric populations [30] and people with known cardiovascular disease (CVD) [70]. In addition, the participants in the current study were not competitive athletes and were from a convenience sample. Therefore, more research is still needed in other populations with differing ages and by selective recruitment of participants with health or training statuses. Second, HRV collection took place under resting conditions in supine and seated positions utilizing a spontaneous breathing rate. Other studies have utilized different positions, such as standing [31], or different conditions, such as pre- or post-exercise [33,70]. Although the body positioning and resting breathing paradigms were chosen based on the most commonly utilized methods of HRV data collection [12,42,48,71], additional investigation regarding different positions, physiological statuses, dynamic scenarios (e.g., exercise), and breathing paradigms are warranted. Third, only PPG-derived time-domain metrics were validated in the current study, even though frequency-domain and non-linear metrics are also commonly utilized throughout the literature [30]. Thus, future research should explore the concurrent validity of PPG sensors of these other HRV metrics. Fourth, while the ECG and HRM devices had similar sampling frequencies (i.e., 1000 Hz), the PPG finger sensor sampled at a lower rate (i.e., 500 Hz). Therefore, the instrument errors associated with differing sampling frequencies may impact the calculation of HRV metrics. That said, from an ecological standpoint, these differing sampling frequencies appeared to have little impact on the test–retest reliability or concurrent validity of the PPG finger sensor based on the findings of the current study, but it is unknown if it would have a larger impact in non-resting conditions, particularly when heart rates are elevated [12]. Fifth, while data were collected across two testing sessions for the test–retest reliability analyses, the study was largely cross-sectional in nature. It is possible that differing test–retest reliability and validity could be identified if data were collected from all devices over the course of a longitudinal period of time. Lastly, the consumption of water and/or food before each testing session was not controlled for across participants, nor were other non-cardiovascular conditions controlled for via exclusion criteria, which could influence HRV measures [38].

## 5. Conclusions

In conclusion, the findings for the current study observed adequate test–retest reliability and concurrent validity of the PPG finger sensor paired with the Elite HRV smartphone application. These results are of particular relevance to those who want to collect resting HRV data in field-based settings. Specifically, sports scientists can utilize the pairing of the PPG sensor with the smartphone application to monitor workload and recovery, guide exercise progression, and enhance the performance of athletes more easily. These findings support the collection of resting short-term HRV in non-laboratory settings by clinicians, researchers, and sports scientists, thus broadening the utilization of HRV across multiple disciplines.

## Figures and Tables

**Table 1:** Participant characteristics stratified by position.

Characteristic	Total Sample (*n* = 44)	Supine (*n* = 38)	Seated (*n* = 41)
Age (yrs)	23.13 ± 4.45	23.05 ± 4.29	23.05 ± 4.09
Sex (%)			
Male	48.9	47.4	48.8
Female	51.1	52.6	51.2
Height (cm)	170.2 ± 9.08	169.40 ± 8.52	170.15 ± 8.98
Body Mass (kg)	74.14 ± 16.50	72.03 ± 14.03	72.83 ± 13.30
BMI (kg/m^2^)	25.39 ± 4.13	25.07 ± 3.70	25.15 ± 3.48

Abbreviations: BMI, body mass index.

**Table 2:** Absolute and relative test–retest reliability of HRV devices in the supine position (*n* = 38).

Device	Day 1 (Mean ± SD)	Day 2 (Mean ± SD)	ICC_2,1_ (95% CI)	SEM	CV (%)	MDC
R-R (ms)						
ECG	936.79 ± 171.00	924.58 ± 168.12	0.860 (0.748, 0.925)	63.35	18.22	175.60
HRM	937.42 ± 171.76	923.89 ± 168.21	0.857 (0.742, 0.923)	64.25	18.26	178.08
PPG Sensor	938.08 ± 173.68	922.24 ± 170.17	0.852 (0.733, 0.920)	66.24	18.48	183.61
HR (bpm)						
ECG	66.00 ± 11.52	66.97 ± 12.28	0.855 (0.738, 0.922)	4.54	17.90	12.58
HRM	66.00 ± 11.49	67.16 ± 12.35	0.853 (0.736, 0.921)	4.57	17.91	12.67
PPG Sensor	66.30 ± 11.50	67.48 ± 12.54	0.847 (0.725, 0.917)	4.71	17.97	13.06
lnRMSSD (ms)						
ECG	3.94 ± 0.58	3.85 ± 0.67	0.842 (0.716, 0.914)	0.25	16.03	0.69
HRM	3.94 ± 0.58	3.86 ± 0.67	0.840 (0.713, 0.914)	0.25	15.97	0.69
PPG Sensor	3.99 ± 0.52	3.99 ± 0.55	0.764 (0.591, 0.870)	0.26	13.34	0.72
lnSDNN (ms)						
ECG	3.92 ± 0.49	3.87 ± 0.48	0.820 (0.680, 0.902)	0.21	12.49	0.57
HRM	3.92 ± 0.49	3.87 ± 0.49	0.815 (0.672, 0.899)	0.21	12.53	0.58
PPG Sensor	4.13 ± 0.41	4.16 ± 0.40	0.604 (0.356, 0.773)	0.25	9.80	0.71

Abbreviations: ECG, electrocardiography; HRM, heart rate monitor; PPG, photoplethysmography; ICC_2,1_, two-way random intraclass correlation coefficient; SEM, standard error of the measure; CV; coefficient of variation; MDC, minimal detectable change; HR, heart rate; lnRMSSD, natural log of root mean square of the successive differences; lnSDNN, natural log of standard deviation of normal R-R intervals.

**Table 3:** Descriptives, bias, error, and agreement between devices in supine position (*n* = 38).

Parameter	PPG Sensor (Mean ± SD)	Criterion (Mean ± SD)	Bias (Mean ± SD)	95% LOA (±2 SD_Bias_)	PE (%)	Effect Size (Hedges’ *g*)
ECG						
R-R (ms)	938.08 ± 173.68	936.79 ± 171.00	1.29 ± 4.53	930.49, 948.24	0.95	0.01 *trivial*
HR (bpm)	66.30 ± 11.50 *	66.00 ± 11.52	0.30 ± 0.57	65.47, 67.72	1.70	0.03 *trivial*
lnRMSSD (ms)	3.99 ± 0.52 *	3.94 ± 0.58	0.05 ± 0.10	3.85, 4.23	4.74	0.09 *trivial*
lnSDNN (ms)	4.13 ± 0.41 *	3.92 ± 0.49	0.21 ± 0.13	4.08, 4.60	6.50	0.47 *small*
HRM						
R-R (ms)	938.08 ± 173.68	937.42 ± 171.76	0.65 ± 4.01	930.88, 946.60	0.84	0.00 *trivial*
HR (bpm)	66.30 ± 11.50 *	66.00 ± 11.49	0.30 ± 0.55	65.52, 67.68	1.64	0.03 *trivial*
lnRMSSD (ms)	3.99 ± 0.52 *	3.94 ± 0.58	0.05 ± 0.09	3.87, 4.23	4.40	0.09 *trivial*
lnSDNN (ms)	4.13 ± 0.41 *	3.92 ± 0.49	0.21 ± 0.13	4.09, 4.59	6.24	0.47 *small*

Abbreviations: ECG, electrocardiography; HRM, heart rate monitor; PPG, photoplethysmography; 95% LOA, 95% limits of agreement; PE, percentage errors; HR, heart rate; lnRMSSD, natural log of root mean square of the successive differences; lnSDNN, natural log of standard deviation of normal R-R intervals. * Significant difference between PPG sensor and ECG or HRM via paired *t*-test (*p* < 0.05).

**Table 4:** Absolute and relative test–retest reliability of HRV devices in the seated position (*n* = 41).

Device	Day 1 (Mean ± SD)	Day 2 (Mean ± SD)	ICC_2,1_ (95% CI)	SEM	CV (%)	MDC
R-R (ms)						
ECG	871.66 ± 146.97	856.59 ± 139.10	0.875 (0.778, 0.931)	50.57	16.55	140.18
HRM	877.63 ± 146.81	859.59 ± 138.18	0.865 (0.761, 0.926)	52.42	16.41	145.31
PPG Sensor	872.78 ± 144.89	855.57 ± 138.92	0.862 (0.756, 0.924)	52.71	16.42	146.11
HR (bpm)						
ECG	70.59 ± 11.25	71.93 ± 11.75	0.885 (0.795, 0.937)	3.89	16.14	10.79
HRM	70.12 ± 11.24	71.61 ± 11.65	0.880 (0.786, 0.934)	3.97	16.15	11.01
PPG Sensor	70.92 ± 10.92	72.25 ± 11.63	0.877 (0.781, 0.932)	3.96	15.75	10.98
lnRMSSD (ms)						
ECG	3.70 ± 0.60	3.73 ± 0.58	0.929 (0.871, 0.962)	0.16	15.87	0.44
HRM	3.70 ± 0.60	3.72 ± 0.59	0.926 (0.866, 0.960)	0.16	15.96	0.45
PPG Sensor	3.88 ± 0.49	3.87 ± 0.48	0.889 (0.802, 0.939)	0.16	12.46	0.45
lnSDNN (ms)						
ECG	3.92 ± 0.48	3.95 ± 0.44	0.907 (0.832, 0.949)	0.14	11.78	0.39
HRM	3.92 ± 0.48	3.95 ± 0.44	0.909 (0.836, 0.951)	0.14	11.66	0.38
PPG Sensor	4.18 ± 0.41	4.20 ± 0.35	0.848 (0.732, 0.916)	0.15	9.12	0.41

Abbreviations: ECG, electrocardiography; HRM, heart rate monitor; PPG, photoplethysmography; ICC_2,1_, two-way random intraclass correlation coefficient; SEM, standard error of the measure; CV; coefficient of variation; MDC, minimal detectable change; HR, heart rate; lnRMSSD, natural log of root mean square of the successive differences; lnSDNN, natural log of standard deviation of normal R-R intervals.

**Table 5:** Descriptives, bias, error, and agreement between devices in seated position (*n* = 41).

Parameter	Finger Sensor (Mean ± SD)	Criterion (Mean ± SD)	Bias (Mean ± SD)	95% LOA (±2 SD_Bias_)	PE (%)	Effect Size (Hedges’ *g*)
ECG						
R-R (ms)	872.78 ± 144.89	871.66 ± 146.97	1.21 ± 8.90	856.26, 891.35	2.00	0.01 *trivial*
HR (bpm)	70.92 ± 10.92 *	70.59 ± 11.25	0.34 ± 0.89	69.51, 73.00	2.47	0.03 *trivial*
lnRMSSD (ms)	3.88 ± 0.49 *	3.70 ± 0.60	0.18 ± 0.21	3.64, 4.48	11.00	0.33 *small*
lnSDNN (ms)	4.18 ± 0.41 *	3.92 ± 0.48	0.26 ± 0.15	4.14, 4.74	7.46	0.58 *small*
HRM						
R-R (ms)	872.78 ± 144.89	877.63 ± 146.81	0.90 ± 8.91	856.22, 854.42	2.00	0.03 *trivial*
HR (bpm)	70.92 ± 10.92	70.12 ± 11.24	0.34 ± 0.95	69.39, 73.12	2.64	0.07 *trivial*
lnRMSSD (ms)	3.88 ± 0.49 *	3.70 ± 0.60	0.17 ± 0.21	3.63, 4.47	11.11	0.32 *small*
lnSDNN (ms)	4.18 ± 0.41 *	3.92 ± 0.48	0.25 ± 0.16	4.12, 4.74	7.63	0.59 *small*

Abbreviations: ECG, electrocardiography; HRM, heart rate monitor; PPG, photoplethysmography; 95% LOA, 95% limits of agreement; PE, percentage errors; lnRMSSD, natural log of root mean square of the successive differences; lnSDNN, natural log of standard deviation of normal R-R intervals. * Significant difference between Finger Sensor and ECG or Polar H10 HRM via paired *t*-test (*p* < 0.05).

## Data Availability

The data presented in this study are available upon request from the corresponding author.

## Supplementary Materials

Supplementary Table: STROBE Statement—Checklist of items that should be included in reports of *cross-sectional studies*. The following supporting information can be downloaded at: https://www.mdpi.com/article/10.3390/sports13020029/s1, STROBE checklist.
